# Cell surface sialylation affects binding of enterovirus 71 to rhabdomyosarcoma and neuroblastoma cells

**DOI:** 10.1186/1471-2180-12-162

**Published:** 2012-08-01

**Authors:** Pei-Yi Su, Yueh-Tung Liu, Hsin-Yueh Chang, Sheng-Wen Huang, Ya-Fang Wang, Chun-Keung Yu, Jen-Ren Wang, Chuan-Fa Chang

**Affiliations:** 1 Department of Medical Laboratory Science and Biotechnology, National Cheng Kung University, No. 1, University Road, Tainan 70101, Taiwan; 2 Institute of Basic Medical Sciences, National Cheng Kung University, No. 1, University Road, Tainan 70101, Taiwan; 3 Center of Infectious Disease and Signaling Research, Medical College, National Cheng Kung University, No. 1, University Road, Tainan 70101, Taiwan; 4 Department of Microbiology and Immunology, National Cheng Kung University, No. 1, University Road, Tainan 70101, Taiwan; 5 National Applied Research Laboratories, National Laboratory Animal Center, No. 128 Academia Road Section 2, Nan-Kang, Taipei 11529, Taiwan; 6 Blood Bank, Kaohsiung Veterans General Hospital, No. 386, Ta-Chung 1st Road, Kaohsiung 81362, Taiwan

**Keywords:** Enterovirus 71, Sialic acid, RD, SK-N-SH, Lectin affinity chromatography

## Abstract

**Background:**

Enterovirus 71 (EV71) is a major causative agent of hand-foot-and-mouth disease (HFMD), and infection of EV71 to central nerve system (CNS) may result in a high mortality in children less than 2 years old. Although there are two highly glycosylated membrane proteins, SCARB2 and PSGL-1, which have been identified as the cellular and functional receptors of EV71, the role of glycosylation in EV71 infection is still unclear.

**Results:**

We demonstrated that the attachment of EV71 to RD and SK-N-SH cells was diminished after the removal of cell surface sialic acids by neuraminidase. Sialic acid specific lectins, *Maackia amurensis* (MAA) and *Sambucus Nigra* (SNA), could compete with EV71 and restrained the binding of EV71 significantly. Preincubation of RD cells with fetuin also reduced the binding of EV71. In addition, we found that SCARB2 was a sialylated glycoprotein and interaction between SCARB2 and EV71 was retarded after desialylation.

**Conclusions:**

In this study, we demonstrated that cell surface sialic acids assist in the attachment of EV71 to host cells. Cell surface sialylation should be a key regulator that facilitates the binding and infection of EV71 to RD and SK-N-SH cells.

## Background

Enterovirus 71 (EV71), an RNA virus of the *Picornaviridae* family, is first recognized from the patients with neurological abnormalities in California in 1969 [1]. It is known to be a causative agent of hand-foot-and-mouth disease (HFMD), and occasionally its infection would lead to severe complications including encephalitis, aseptic meningitis, pulmonary edema or hemorrhage, and acute flaccid paralysis [2]. Outbreaks of EV71 had been reported worldwide during the last decade [2-7]. In Taiwan, there was a large epidemic of HFMD in 1998. More than 120,000 cases were reported and the outbreak resulted in 78 deaths [2]. Two years later, there was another outbreak of HFMD with 80,677 reports and 41 deaths (data from CDC, Taiwan). EV71 can induce the apoptosis of human glioblastoma cells [8], human microvascular endothelial cells [9], and Jurkat cells [10]. Although it has been demonstrated that the spinal cord and brain stem were the target of EV71 infections [6,11], the infection mechanism, tissue tropism, and the neurovirulence of EV71 remain unclear.

In 2009, two receptors for EV71 were discovered [12,13]. Nishimura et al. found that human P-selectin glycoprotein ligand-1 (PSGL-1) was a functional receptor for EV71 [12]. Yamayoshi et al. reported that scavenger receptor class B2 (SCARB2) was cellular receptor for EV71 [13]. PSGL-1 is glycosylated with sialyl Lewis^x^ epitope, and SCARB2 is also a highly glycosylated protein. According to these results, cell surface glycans should participate in the infection of EV71. Hence, the glycomic factors which contribute to the epidemics of EV71 infection have attracted our attention.

Carbohydrates expressed on cell surface involve in many physiological and pathological communications by interacting with their corresponding proteins or receptors [14,15]. Among these events, cell surface glycan receptors which mediate viral binding and infection were well documented. For instance, Jackson et al. indicated that the entry of food-and-mouse disease virus (FMDV) into cell was initiated by the contact with cell surface heparin sulfate [16]. Sulfated polysaccharides extracted from sea algae were demonstrated having potential to prevent the infection of viruses including herpes simplex virus (HSV), cytomegalovirus (CMV), human immunodeficiency virus (HIV) and enterovirus [17-21]. Lactoferrin, an 80 kDa iron binding glycoprotein presented in several mucosal secretions [22,23], was reported to inhibit interaction between EV71 VP1 to RD cells [24,25]. In addition, sialic acids were cell surface ligands for many hemagglutinins (HAs) or viral proteins (VPs) including influenza, parainfluenza, reovirus type3, adenovirus type 37, human rhinovirus 87, human enterovirus type 70 [26], coxsackievirus A24 [27], and hepatitis A virus [28].

Since the role and function of surface glycans in the attachment and infection of EV71 is still vague, this paper aims to decipher these issues and figure out the most important glycomic constituents. Two EV71 susceptible human cell lines, rhabdomyosarcoma cells (RD cells) and human neuroblastoma cells (SK-N-SH cells), are subjected to virus binding assay. Cells were pretreated with neuraminidase or α2-3/α2-6 sialic acid binding lectins (MAA/SNA) for revealing the role of cell surface sialic acids during EV71 attachment. In addition, fetuin (a highly sialylated glycoprotein) was subjected to validate the interaction of sialic acids with EV71. The significance of sialylation on SCARB2 was also evaluated.

## Results

### Role of sialylation in EV71 infection

**Figure 1:**
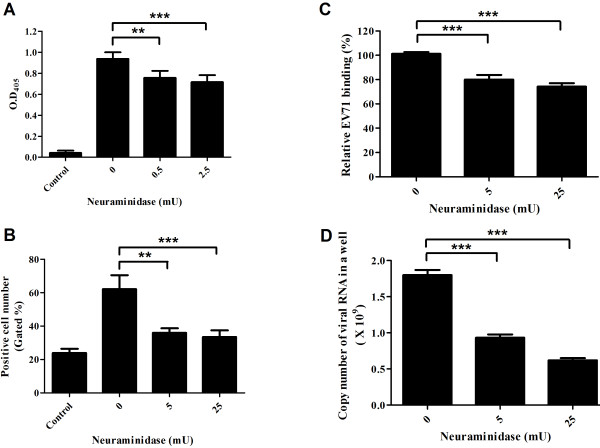
**The attachment and infection of EV71 to RD cells are affected by neuraminidase treatment.** Cells were pretreated with neuraminidase followed by infection with EV71 MP4. The bound virus was analyzed by ELISA, flow cytometry and real-time PCR. The binding of virus to RD cells treated with different units of neuraminidase was reduced by 20% and 32% measured by ELISA (**A**), by 27% and 29% measured by flow cytometry (**B**), and by 20% and 27% measured by real-time PCR (**C**). The replication of EV71 dropped by 49% and 66% in neuraminidase treated cells measured by analyzing the copy number of EV71 RNA using real-time PCR after 24 hours incubation (**D**). **: *P* < 0.01; ***: *P* < 0.001 (two-tailed test). Each of the results was averaged from at least six independent assays.

**Figure 2:**
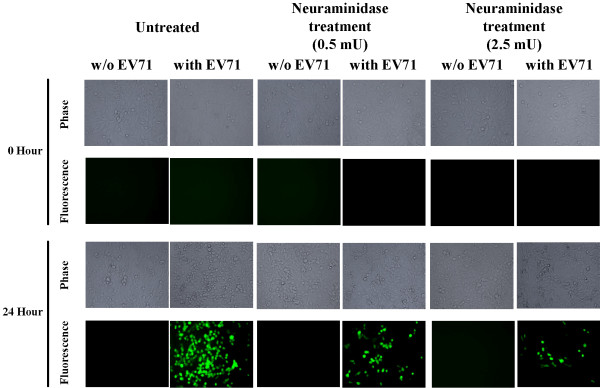
**The infection and replication of EV71 to RD cells are affected by neuraminidase treatment investigated with EV71-GFP infection.** Cells pretreated with or without neuraminidase (5 mU and 25 mU) were infected with or without EV71-GFP. The cell number, CPE, and fluorescence intensity were observed by fluorescence microscope. After 48 hours, higher fluorescence intensity was found in untreated cells than neuraminidase pretreated cells.

**Figure 3:**
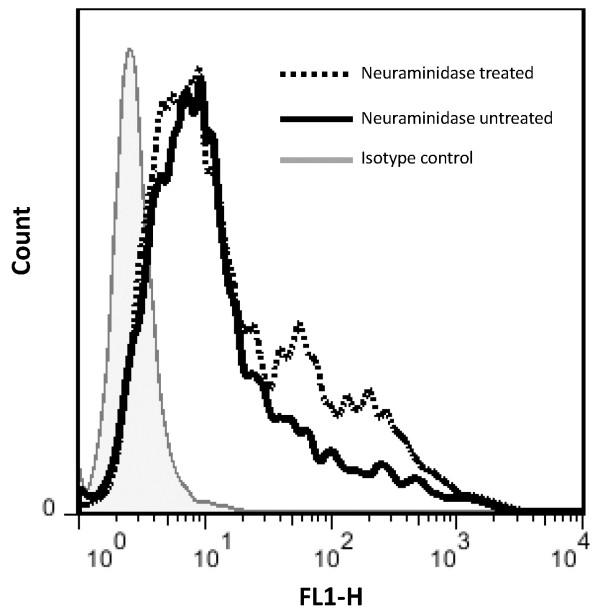
**The expression of RD cell surface SCARB2 with or without neuraminidase treatment measured by flow cytometry.** Cell surface SCARB2 was nearly the same after 25 mU of neuraminidase treatment.

Since sialic acids participated in the attachment of many viruses of the *Picornaviridae* family [28,29], we verified the effects of sialic acids in EV71 infection. RD cells pretreated with different units of neuraminidase were subjected to the binding of EV71 by ELISA, flow-cytometry and real-time PCR assay. We found that the binding of EV71 to RD cells decreased dramatically in a dose dependent manner, which was accompanied with the increasing units of neuraminidase treatment (19-24% in ELISA assay, 42-46% in flow cytometry; 21-27% in real-time PCR and 48-66% in real-time PCR assay after 24 hours incubation; Figure 1 A-D). A clear cytopathic effect was also observed along with the decrease of neuraminidase used in EV71-GFP infected RD cells (Figure 2). It should be noted that the expression of cell surface SCARB2 was nearly the same after neuraminidase treatment (Figure 3).

**Figure 4:**
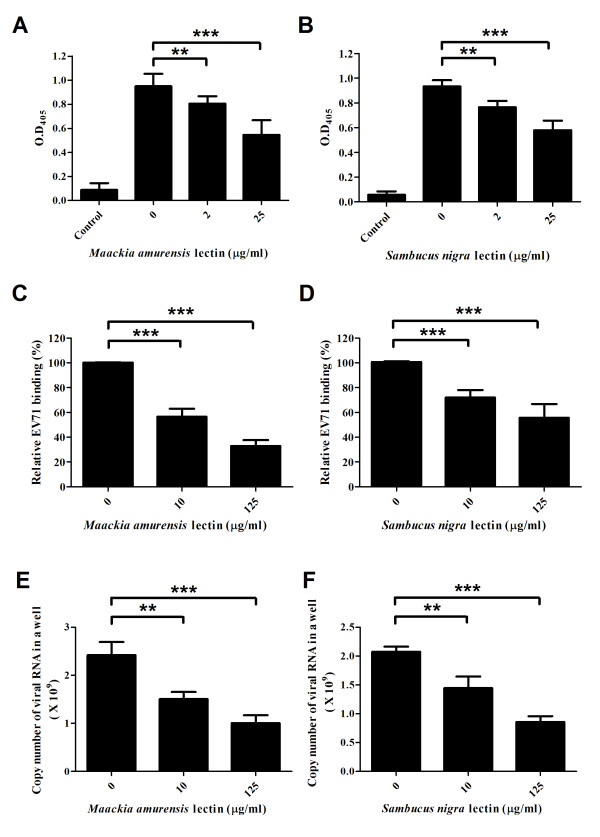
**The attachment and infection of EV71 to RD cells are affected by sialic acid specific lectin treatment.** Cells were preincubated with MAA (*maackia amurensis*) or SNA (*sambucus nigra*) followed by infection with EV71 MP4. The bound EV71 was analyzed by ELISA and real-time PCR, and the subsequent replication of EV71 in RD cells was detected by real-time PCR analysis. The binding of virus to RD cells treated with different concentrations of MAA was reduced by 19% and 45% measured by ELISA (**A**) and by 37% and 68% measured by real-time PCR (**C**). The replication of EV71 dropped 38% and 59% after MAA treatment measured by real-time PCR after 24 hours incubation (**E**). The virus binding of SNA treated cells reduced by 18% and 38% measured by ELISA (**B**), and by 28% and 45% measured by real-time PCR (**D**). The replication of EV71 dropped 30% and 58% after SNA treatment measured by RT-PCR after 24 hours incubation (**F**). **: *P* < 0.01; ***: *P* < 0.001 (two-tailed test). Each of the results was averaged from at least six independent assays.

**Figure 5:**
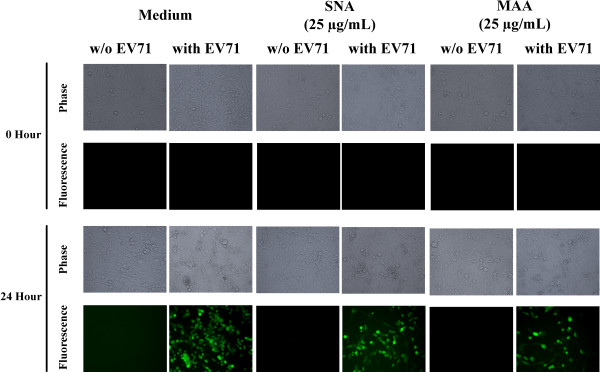
**The infection and replication of EV71 to RD cells are affected by lectin treatment investigated with EV71-GFP infection. Cells preincubated with or without MAA/SNA were infected with or without EV71-GFP.** The cell number, CPE, and fluorescence intensity were observed by fluorescence microscope. After 48 hours, higher fluorescence intensity was found in untreated cells than neuraminidase pretreated cells.

**Figure 6:**
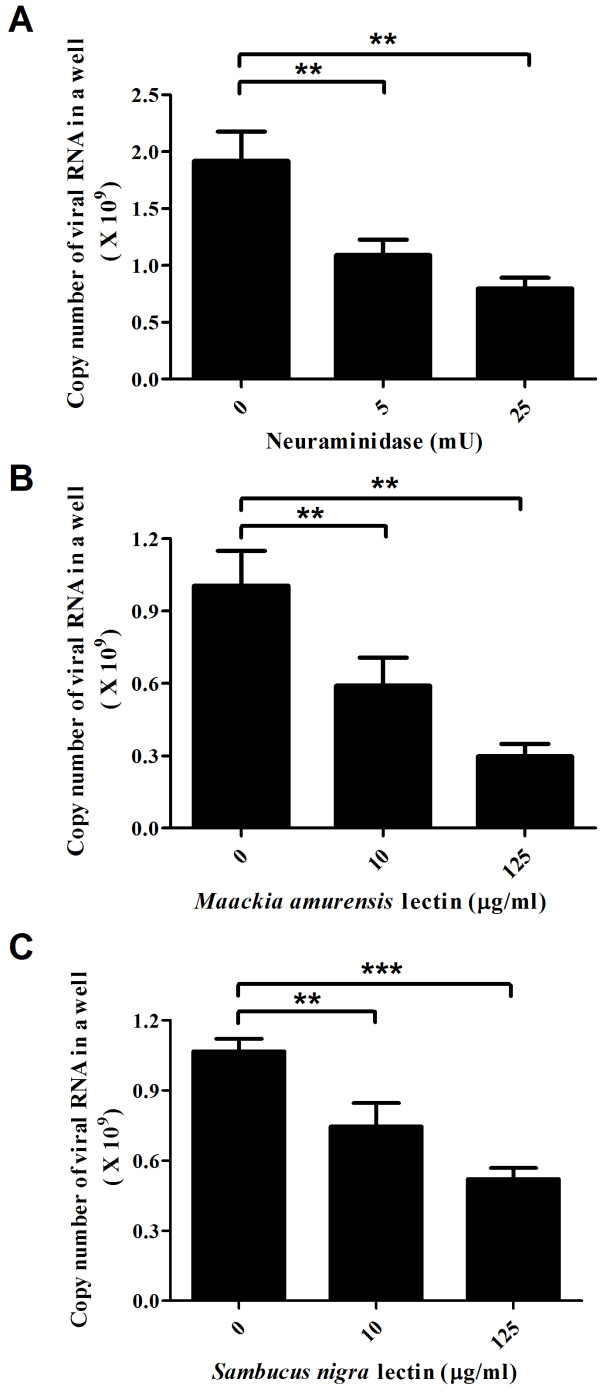
**The attachment and infection of EV71 4643 to SK-N-SH cells are affected by neuraminidase or sialic acid specific lectin treatment. SK-N-SH cells were pretreated with neuraminidase, MAA or SNA before infected with EV71 4643.** (**A**) The copy number of EV71 dropped 44% and 59% in neuraminidase treated cells. (**B**) The copy number of EV71 reduced by 42% and 59% in MAA treated cells. (**C**) The copy number of EV71 decreased by 31% and 52% in SNA treated cells. **: *P* < 0.01; ***: *P* < 0.001 (two-tailed test). Each of the results was averaged from at least six independent assays.

Based on these results, we further investigated the sialic acid linkage preference of EV71 by lectin competition assay and carbohydrate solution microarray [30]. MAA preferentially recognized α2-3 linked sialosides and SNA specifically interacted with α2-6 linked sialosides. As shown in Figure 4 A-F, preincubation of RD cells with MAA or SNA reduced the interactions of EV71 to RD cells up to 68% in a dose dependent manner. The retarded cytopathic effect also indicated that the replication of EV71-GFP in RD-cells was decreased by lectin treatment (Figure 5). These findings demonstrated that EV71 may interact with both α2-3 and α2-6 linked sialylated glycoproteins on RD cell surface. Additionally, the same results and inhibition trends were obtained when we applied the same assays on SK-N-SH cells which were infected with EV71 4643 (X, Y, and Z% in real-time PCR assays; Figure 6 A-C).

**Figure 7:**
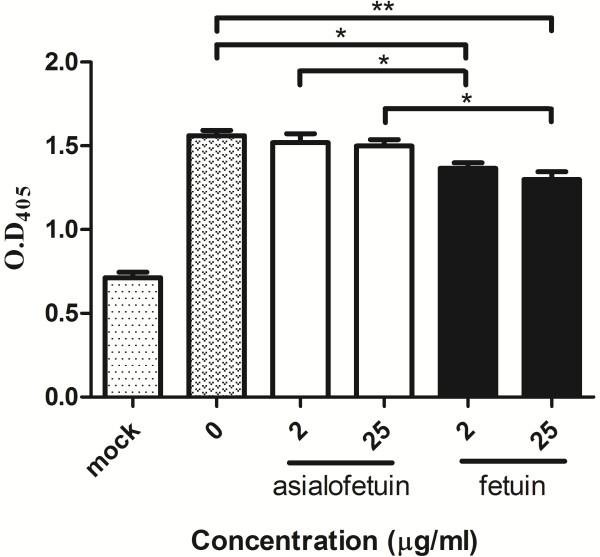
**Fetuin blocks the attachment of EV71 to RD cells. Cells were preincubated with fetuin or asialofetuin and infected with EV71.** Asialofetuin showed no effect on virus binding, but the attachment of EV71 to RD cells decreased by 12% to 14% in fetuin preincubated cells. *: *P* < 0.05; **: *P* < 0.01 (two-tailed test). Each of the results was averaged from at least seven independent assays.

Because it has been reported that lactoferrin, a highly sialylated glycoprotein, can inhibit the infection of EV71 [24,25], we used another highly sialylated glycoprotein to confirm these interactions between EV71 with sialic acid. Fetuin and asialofetuin were subjected to EV71 binding assay. Not surprisingly, pretreated cells with fetuin reduced the attachment of EV71 to RD cells by 12-14% (statistically significant, Figure 7). These findings encouraged us to identify the carbohydrate ligands for EV71 viral particles and VP1 protein (recombinant protein from *E. coli*) by glycan solution microarray. But, unfortunately, none of the binding signals were observed (Additional file 1 Supplementary information).

### Characterization of SCARB2 sialylation in EV71 infection

**Figure 8:**
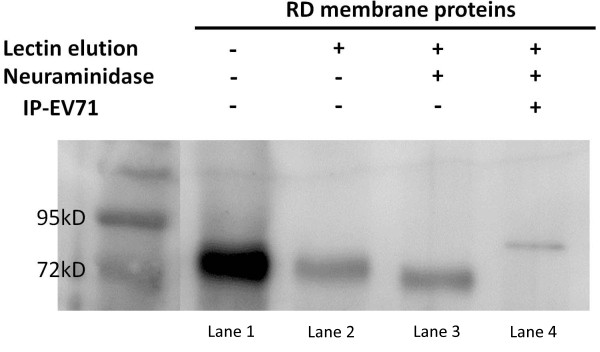
**Western blotting of glycoprotein fractions with anti-SCARB2 antibody.** Lane 1: RD cell membrane extracts; Lane 2: MAA/SNA lectin affinity chromatography purified sialylated glycoproteins; Lane 3: desialylated glycoproteins; Lane 4: desialylated glycoproteins immunoprecipitated with EV71 MP4. All of the purified proteins were subjected to western blotting and stained by anti-SCARB2 antibody. SCARB2 could be observed in all of the fractions. Band in lane 3 was slightly shifted down after neuraminidase treatment. But, owing to non-reducing condition, band in lane 4 was slightly shifted up compared to band in lane 3.

**Figure 9:**
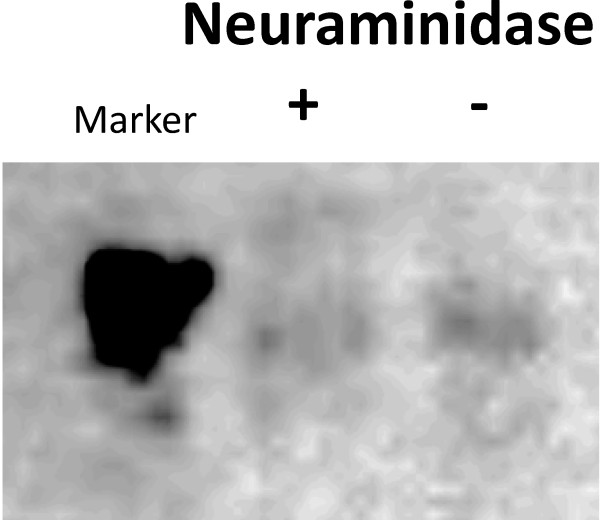
**Interactions between recombinant hSCARB2 with EV71 are reduced after desialylation.** The binding is detected by Viral-Overlaying Protein Binding Assay (VOPBA) with anti-EV71 antibody and HRP conjugated anti-mouse antibody on LAS-3000.

Based on these findings, we tried to look deep inside the relationships of sialylation with viral receptor. By using lectin affinity chromatography (LAC) which contained MAA and SNA-agarose beads, we purified sialylated membrane proteins from RD cell membrane extracts. Desialylation was performed with neuraminidase on purified glycoproteins to remove sialic acids. The desialylated glycoproteins were subjected to immunoprecipitation assay, in which EV71 viral particles were immobilized on protein G agarose beads through anti-EV71 antibody. As shown in Figure 8, the cellular receptor of EV71, SCARB2, was observed in all of the purified and immunoprecipitated protein fractions. Because of the neuraminidase treatment, band in lane 3 was slightly shifted down. In addition, band in lane 4 was slightly shifted up owing to the non-reducing treatment of EV71 pulled down fractions. To determine whether sialylation on SCARB2 contribute to its interaction with EV71, the binding of EV71 to recombinant human SCARB2 (hSCARB2, with or without neuraminidase treatment) was analyzed by virus overlay protein binding assay (VOPBA). The result showed that desialylation of hSCARB2 curtailed the binding ability with EV71 (Figure 9).

## Discussion

Glycans that expressed on cell surface are involved in cell-cell adhesion, leukocyte rolling, cell-extracellular matrix interaction, and microbes’ infection [31-33]. Carbohydrates, especially sialic acids, are also reported as receptors for gram positive or negative bacteria, viruses, protozoa, and plant lectins [28]. For example, sialyl Lewis^x^ is a ligand for the SabA protein of *Helicobacter pylori*[34]. Cholera toxin of *Vibrio chlolerae* specifically binds to the GM1 moiety [35]. Human influenza virus recognizes α2-6 sialylated glycans and infects host cells subsequently [36,37]. Glycosaminoglycan, such as hyaluronic acid and chondroitin sulfate, are confirmed as antiviral agents in preventing Coxsackievirus B5 and dengue virus, respectively [38,39]. Further, sialic acid is reported as receptors of many *Picornaviridae* viruses [28,29].

Several methods were established to evaluate the attachment and reproduction efficiency of EV71. ELISA assay and flow cytometry provided reliable and reproducible results in quantifying bound EV71 viral particles on cell surface. The binding and subsequent replication of EV71 was detected by measuring the copy number of viral RNA by real-time PCR. In addition, the infection and replication of EV71 could also be confirmed by observing the fluorescence intensity and cytopathic effects in EV71-GFP infected cells. RD is an EV71 highly susceptible cell line which has been applied for viral replication. SK-N-SH cells established from human neuroblastoma were cell model for investigating the EV71 caused neuron toxicity. RD and SK-N-SH cells were infected with EV71 MP4 (mouse adapted strain) and EV71 4643 (human clinical isolates), respectively [40].

Since glycosylation was a common and significant feature for cellular and functional receptors of EV71, we first investigated the effects of tunicamycin and benzyl-α-GalNAc (inhibitor for protein *N*- and *O*-glycosylation, respectively) in the binding and infection of EV71 to RD cells. Both of the inhibitors decreased the binding of EV71 to RD cells significantly (data not shown). We thus investigated whether the terminal sialic acids on cell surface was involved in EV71 infection to RD and SK-N-SH cells. We found that the expression of cell surface SCARB2 was slightly increased after neuraminidase treatment, and neuraminidase treatment reduced virus binding to RD and SK-N-SH cells in a dose-dependent manner. In addition, the replication of virus was decreased because the binding of EV71-GFP to RD cells was reduced after neuraminidase treatment. These results indicated that sialylation on cell surface should be involved in the attachment and infection of EV71.

As long as there are two major glycosidic linkages between sialic acid with galactose, we applied the lectin competition assay to characterize the binding preference of EV71 to RD and SK-H-SN cells. Not surprisingly, the binding of EV71 was restrained by both lectins on RD and SK-H-SN cells. Both cell surface α2-3- and α2-6-linked sialosides were participated in the binding of EV71 to host cells. The replication of virus was also dropped because the interaction of EV71-GFP to RD cells was blocked by MAA or SNA. These observations can also be found in the infection of other *Picornaviridae* viruses such as human rhinovirus 87, encephalomyocarditis virus, and hepatitis A virus [28]. Then, fetuin/asialofetuin blocking assay was performed and the result indicated that sialylated glycoproteins, such as fetuin, lactoferrin and milk proteins, were inhibitors of EV71 infection [24,25,29]. In order to further identify the carbohydrate epitopes for EV71 infection, viral particles and recombinant viral capsid protein were subjected to carbohydrate solution microarray analysis. But, we could not observe any positive binding signal for viral particles or recombinant VP1 protein. It might be because we don’t have sufficient sialylated epitopes in our microarray library. Further investigations are in progress (collaborate with CFG).

To further characterize the role of sialylation on EV71 cellular receptor, we isolated cell membrane sialylated glycoproteins by lectin affinity chromatography. LAC was a common and useful tool for proteomic and glycomic analysis [41-45]. For instance, Butterfield et al. enriched and analyzed abnormal glycoproteins from brain of Alzheimer disease patient by using LAC [41]. Alvarez-Manilla and colleagues also identified potential glycobiomarkers from embryonic stem cells with LAC technology [43]. Hence, sialylated membrane proteins were purified with MAA/SNA lectin-agarose column from RD cell membrane extractions. Then, the purified glycoproteins were treated with neuraminidase to remove the effect of sialic acid. The desialylated glycoproteins were subjected to immunoprecipitation assay that pulled down proteins specifically interacted with EV71. Not surprisingly, SCARB2 was observed in western blotting of LAC purified fraction, neuraminidase treated fraction, as well as the EV71 immunoprecipitated fraction. It should be noted that the position of band in lane 4 (EV71 immunoprecipitated fraction) was inconsistent with band in lane 3. This result may come from sample preparation under non-reducing condition which could not break SCARB2-viral protein interactions. These observations prompted us to investigate the binding of EV71 to sialylated and desialylated SCARB2. By using VOPBA, we found that recombinant hSCARB2 lost some of the binding ability to EV71 after desialylation. The same phenomenon have been observed by Yamayoshi et al who found that the interaction of EV71 with recombinant hSCARB2 was moderately decreased after removing *N*-glycans from hSCARB2 by enzymatic hydrolysis [46]. Taken together, all of the results indicated that the attachment of EV71 to cell surface receptor should be assisted with sialic acids.

## Conclusions

Based on our findings, we concluded that cell surface sialylation was important for EV71 infection to RD and SK-N-SH cells. Although the glycan epitopes for EV71 was still unclear, these evidences sufficiently supported that sialylation of cell surface glycoproteins could assist the attachment of EV71 to host cells. In addition, we also demonstrated that SCARB2 was a sialylated glycoprotein. Interactions between SCARB2 with EV71 were decreased after desialylation. Our findings not only demonstrated the important role of sialic acid in EV71 infection to RD and SK-N-SH cells, but also opened a new direction for anti-EV71 drug discovery. Finally, identification and characterization of glycans or proteins which interact with EV71 are now in progress.

## Methods

### Virus amplification and purification

RD and SK-N-SH cells (ATCC, Manassas, VA) were maintained in Dulbecco’s modified Eagle’s medium (DMEM) contained 10% fetal bovine serum (FBS), 2.0 mM L-glutamine, 100 IU of penicillin, and 100 μg of streptomycin. The infectious clone of mouse-adapted EV71 MP4, 4643 and EV71-GFP were obtained from Dr. Jen-Ren Wang. GFP was located in the replicon between P2 and P3 nonstructural regions. In vitro transcription of the linear plasmid of MP4, 4643 and EV71-GFP were performed by kit (Promega) and purified mRNA was transfected to RD cells (for MP4 and EV71-GFP) or SK-N-SH (for 4643). Virus was amplified in RD cells or SK-N-SH and cultivated using DMEM with 2% FBS at 37°C for 16 to 24 hours. To prepare virus stocks, viruses will be propagated for one more passage in cells. Working stocks contain 10^8^ PFU per ml. The culture medium and cells were collected for purification when CPE was observed. All of the viruses were precipitated with poly ethylene glycol (PEG) and purified by sucrose gradient ultracentrifugation.

### Preparation of EV71 specific monoclonal antibody (1 G3)

The hybridoma cells which produced monoclonal antibody (1 G3) against EV71 VP1 protein region was a gift from Dr. Chun-Keung Yu. The hybridoma cells (10^6^) were injected intraperitoneally into 10-weeks old BALB/c mice after pristane injection. Ascites was collected and the 1 G3 monoclonal antibody was purified by protein-A affinity column on AKTA prime plus (GE Healthcare). The titer of antibody was quantified by ELISA assay.

### General procedures for virus binding assay

#### ELISA

Cells were seeded in 96 microtiter plate and cultured with DMEM containing 10% FBS at 37°C for 72 hours. EV71 MP4 (M.O.I = 100) or EV71 GFP were added into the treated or untreated cells and incubated at 4°C for 3 hours. The reactions were mixed gently every 30 minutes. After wash, the cells were fixed with 4% paraformaldehyde and incubated with anti-EV71 antibody 1 G3 at room temperature for 2 hours. Alkaline phosphatase conjugated anti-mouse IgG (Sigma) was added and incubated at room temperature for 2 hours. After wash, substrate (*p*-nitrophenyl phosphate) solution was added and incubated at room temperature for 30 minutes. The reactions were quenched by adding NaOH (3.0 N) and measured the absorbance at 405 nm by EnVison^TM^ 2103 Multilabel reader (PerkinElmer).

#### Flow cytometry

Treated and untreated cells (4 × 10^5^/assay) harvested from culture plate were washed with PBS once and incubated with EV71 MP4 (M.O.I = 100) at 4°C for 3 hours. After wash, the cells were fixed with 4% paraformaldehyde and incubated with anti-EV71 antibody 1 G3 at room temperature for 2 hours. Alexa 488 conjugated anti-mouse IgG (Invitrogen) was added into the reaction and incubated at 4°C for 1 hour. The histograms of bound viruses were analyzed by FACSCalibur flow cytometer (BD Biosciences).

#### Real-time PCR

Cells were seeded in 6 well plate (2.5 × 10^5^/ well) and cultured with DMEM containing 10% FBS at 37°C for 72 hours. Treated and untreated cells were incubated with EV71 MP4 or 4643 (M.O.I = 10) at 4°C for 1 hour. The total RNA was extracted by RNeasy protect bacteria mini kit (QIAGEN) and the copy number of viral RNA was measured by using LightCycler RNA Master HybProbe kit (Roche). The copy number of viral RNA was calculated using a standard curve. The replication of EV71 was also measured by real-time PCR. Treated and untreated cells were incubated with EV71 MP4 or 4643 (M.O.I = 1) at 4°C for 1 hour. After the unbounded virus was removed, culture medium was added into the well and incubated at 37°C for 24 hours. The total RNA was measured as described above.

#### EV71-GFP infection assay

RD cells were seeded in 96 well plate (1 × 10^4^/ well) and cultured with DMEM containing 10 % FBS at 37°C for 72 hours. Treated and untreated cells were incubated with EV71-GFP (M.O.I = 15) at 37°C for 1 hour. After the unbounded virus was removed, culture medium was added was added into the well and incubated at 37°C for 48 hours. The cell number, CPE, and fluorescence intensity were observed by fluorescence microscope at 0, 24 and 48 hours.

### General procedures for inhibition assays

All of the inhibition assays were performed by treating cells with inhibitors, enzyme, or lectins before EV71 infection. Virus was incubated with cells at 4°C for 3 hours in binding assay, and worked at 37°C for 3 hours in virus infection assay. The bound EV71 was analyzed by ELISA, flow cytometry and real-time PCR followed the procedures mentioned above. The copy number of EV71 was detected by real-time PCR analysis.

#### Inhibitor treatment

Cells were incubated with 0.5 mg/ml tunicamycin (Sigma) or 3.0 mM benzyl-α-GalNAc (Toronto Research Chemicals Inc.) at 37°C for 24 or 48 hours, respectively. After wash, the cells were subjected to virus infection.

#### Neuraminidase treatment

Cells were incubated with 0.5 to 25 mU of neuraminidase (Roche, 11080752001) with 4 mM CaCl_2_ in serum-free DMEM at 37°C for 3 hours followed by wash and EV71 infection. For detecting cell surface SCARB2, the neuraminidase treated cells (10 mU) were incubated with mouse anti-SCARB2 antibody (1:100) and FITC-conjugated goat anti-mouse antibody (1:500) at 4°C for 30 minutes. After wash for three times, the cells were analyzed by FACS caliber with Cell Quest Pro software (BD Biosciences).

#### Lectin competition

Cells were incubated with 2 to 125 μg/ml of MAA (*maackia amurensis*) or SNA (*sambucus nigra*) at 4°C for 30 minutes. After wash, the cells were subjected to virus infection.

#### Fetuin and asialofetuin treatment

RD cells (2x10^4^) were incubated with 2/25 μg/ml of fetuin or asialofetuin at 4°C for 30 minute followed by wash and EV71 MP4 infection (M.O.I = 100). The binding of EV71 was measured by ELISA assay.

### Isolation of cell membrane glycoproteins and sialylated proteins

RD cells were harvested and homogenized in ice-cold homogenization buffer (20 mM Tris–HCl, pH 7.5, 2.0 mM EDTA, 1.0 mM DTT and protein inhibitor cocktail) by using sonicator (Chrom Tech). Cell lysates were obtained by centrifugation and cell pellet was resolved in homogenization buffer. The collected membrane fractions from centrifugation were resuspended in homogenization buffer and analyzed by western blotting. Then, membrane protein fractions were subjected to lectin affinity chromatography that was packaged with SNA and MAA agarose beads (EY Laboratories). The sialylated glycoproteins were eluted by 20 mM ethylenediamine and all of the fractions were collected for further characterization and analyzed by western blotting with anti-SCARB2 monoclonal antibody.

### Immunoprecipitation assay

The purified sialylated glycoproteins were incubated with 5 units of neuraminidase at 4°C for 16 hours. The reaction mixture was transferred to an eppendorf which contained EV71 viral particles, anti-EV71 antibody, and protein G agarose beads. The reaction was incubated at 37°C for 12 hours and the bound proteins were pulled down by centrifugation. After unbound proteins were removed, the agarose beads were washed with PBS buffer for three times and added glycin-HCl (pH 2.0) to break the bindings. The reaction solution was centrifuged to remove Protein A agarose beads and the bound glycoproteins were concentrated and analyzed by western blotting with anti-SCARB2 monoclonal antibody.

### Interactions of EV71 to recombinant hSCARB2 - Viral-Overlaying Protein Binding Assay (VOPBA)

Recombinant h-SCARB-2 protein was purchased from *Abscience* (11063-H03H). 25 μg of the h-SCARB-2 proteins were pretreated with or without neuraminidase (10 mU, Roche, 11080752001) at 37°C. After 17 hours, the proteins were subjected to 10% polyacrylamide gel electrophoresis under non-reducing conditions and transferred to nitrocellulose membrane which was block with binding buffer (1% BSA, 154 mM NaCl, 0.05% Tween-20, 1 mM CaCl_2_) at 4°C for 16 hours. The membrane was incubated with EV71 in binding buffer at 4°C for 16 hours with gentle rocking. After washed three times with binding buffer, the membrane was incubated with anti-virus antibody (1:2000, Millipore, Mab979) at room temperature for 2 hours. HRP conjugated goat anti-mouse IgG antibody (1:5000) was then added, incubated at room temperature for 1 hour and washed by binding buffer for three times. The images were captured by Fujifilm LAS-3000.

### Western blotting

15 μg of h-SCARB-2 proteins were pretreated with or without neuraminidase (10 mU, Roche, 11080752001) at 37°C. After 17 hours, the proteins were denatured in 95°C for 10 min and subjected to 10% polyacrylamide gel electrophoresis. Then, the proteins were transferred to nitrocellulose membrane and blocked with 5% milk with PBS-T at room temperature for 1 hour. The membrane was incubated with anti-SCARB-2 antibody (Abcam, ab106519) at 4°C for 16 h with gentle rocking, and incubated with HRP-conjugated goat anti-mouse IgG antibody at room temperature for 1 hour. The images were analyzed by Fujifilm LAS-3000.

### Statistical analysis

Statistical analysis was performed using student’s T-test for determination of statistical significance. The value of *P < 0.05* was considered to indicate statistical significance. (*: *P* < 0.05; **: *P* < 0.01; ***: *P* < 0.001).

## Abbreviations

EV71: Enterovirus 71; HFMD: Hand-foot-and-mouth disease; PSGL-1: P-selectin glycoprotein ligand-1; SCARB2: Scavenger receptor class B: member 2; RD cells: Rhabdomyosarcoma cells; SK-N-SH cells: Human neuroblastoma cells; MAA: *Maackia amurensis*; SNA: *Sambucus Nigra*; LAC: Lectin affinity chromatography.

## Competing interests

The authors have declared that no competing interests exist.

## Authors’ contributions

Miss Yueh-Tung Liu produced EV71 MP4 and EV71-GFP viruses, and performed the assays including flow cytometry, real-time PCR, and EV71-GFP infection. Miss Pei-Yi Su accomplished the purification and analysis of cell membrane proteins from RD cell lysates, western blotting, and characterization of SCARB2. Miss Liu and Miss Su contributed equally in this work. Miss Hsin-Yueh Chang was in charge of cell culture and the infection assays of EV71 4643 to SK-H-SN cells. Mr. Sheng-Wen Huang established the infectious clones of virus strains. Dr. Ya-Fang Wang developed the mouse adapted EV71 strain (MP4). Dr. Chun-Keung Yu and Dr. Jen-Ren Wang helped in the study design, analysis of the results and preparation of the manuscript. Dr. Chuan-Fa Chang conceived of the study, participated in its design and coordination and wrote the manuscript. All authors read and approved the final manuscript.

## Supplementary Material

Additional file 1 Supplementary information. Click here for file
