# Supplementary material for: Cell surface sialylation affects binding of enterovirus 71 to rhabdomyosarcoma and neuroblastoma cells
Source: BMC Microbiol. 2012 Aug 1;12:162. doi: 10.1186/1471-2180-12-162 (PMC3478995; doi:10.1186/1471-2180-12-162)

Supplementary information

Glycan solution microarray analysis

All of the procedures and incubations must be carried out in the dark. Donor beads (500 ng/well) and biotin-PAA-sugars (20 ng/well, total 88 glycans) mixed with EV71 MP4 (10 μg/well) were incubated at ambient temperature for 1 hour (total 15 μL). The mixture of acceptor beads (500 ng/well), mouse anti-EV71 antibody (50 ng/well), and rabbit anti-mouse IgG antibody (25 ng/well) was added into the reaction to a final volume of 25 μL. After 2 hours incubation at ambient temperature, the binding signals were obtained on the PerkinElmer Envision instrument using the AlphaScreen™ program. The glycans used in this assay were listed in **supplementary information Table 1.** The results were presented by fluorescence intensities.

Supplementary information Table 1

List of the 88 PAA-glycans used in glycan solution microarray.

Supplementary information Figure 1

The binding profile of EV71 viral particles with 88 biotin-PAA-sugars. The binding specificities are indicated by fluorescence intensities (*y*-axis). The sugar identities are designated by numbers (*x*-axis) and shown in detail in **supplementary information**

Table 1. Each of the results was averaged from at least three independent assays.

Supplementary information Figure 2

The binding profile of VP1 protein with 88 biotin-PAA-sugars. The binding specificities are indicated by fluorescence intensities (*y*-axis). The sugar identities are designated by numbers (*x*-axis) and shown in detail in **supplementary information Table 1.** Each of the results was averaged from at least three independent assays.

Supplementary information
Table 1

S-1	Blank-PAA-biotin	S-46	Neu5Acα2-6Galβ-PAA-biotin
S-2	β-GlcNAc-sp-biotin	S-47	Neu5Gcα2-6GalNAc-PAA-biotin
S-3	α-Mannose-PAA-biotin	S-48	Neu5Acα2-3GalNAcα-PAA-biotin
S-4	β-GlcNAc-PAA-biotin	S-49	Blood Group A-tri-PAA-biotin
S-5	β-GalNAc-PAA-biotin	S-50	Blood Group B-tri-PAA-biotin
S-6	α-L-Fuc-PAA-biotin	S-51	H(type2)-PAA-biotin
S-7	α-Neu5Ac-PAA-biotin	S-52	Le^a-PAA-biotin
S-8	α-Neu5Ac-$OCH_2C_6H_4$-p-$NHCOOCH_2$-PAA-biotin	S-53	Le^x-PAA-biotin
S-9	MDP(muramyl dipeptide)-PAA-biotin	S-54	Le^d(H type1)-PAA-biotin
S-10	α-Neu5Gc-PAA-biotin	S-55	3'Sialyl-Lactose-PAA-biotin
S-11	β-D-Gal-3-sulfate-PAA-biotin	S-56	6'Sialyl-Lactose-PAA-biotin
S-12	β-D-GlcNAc-6-sulfate-PAA-biotin	S-57	3-HSO_3-Le^x-PAA-biotin
S-13	GalNAcα1-3Galβ-PAA-biotin	S-58	3-HSO_3-Le^a-PAA-biotin
S-14	Galα1-3Galβ-PAA-biotin	S-59	Galα1-4Galβ1-4Glcβ-PAA-biotin
S-15	Fucα1-2Galβ-PAA-biotion	S-60	Galα1-3Galβ1-4Glcβ-PAA-biotin
S-16	Le^c(Galβ1-3GlcNAc)-PAA-biotin	S-61	GlcNAcβ1-2Galβ1-3GalNAcα-PAA-biotin
S-17	Galβ1-4Glcβ-PAA-biotin (Lactose)	S-62	Neu5Acα2-3Galβ1-4GlcNAcβ-PAA-Biotin
S-18	LacNAc-PAA-biotin	S-63	3'Sialyl-Le^c-PAA-biotin
S-19	Fucα1-3GlcNAcβ-PAA-biotin	S-64	Galα1-3Galβ1-4GlcNAcβ-PAA-biotin, sp=-$NHCOCH_2NH$-
S-20	Fucα1-4GlcNAcβ-PAA-biotin	S-65	GlcNAcα1-3Galβ1-3GalNAcα-PAA-biotin
S-21	GalNAcα1-3GalNAcα-PAA-biotin	S-66	GlcNAcβ1-3Galβ1-3GalNAcα-PAA-biotin
S-22	Galα1-3GalNAcα-PAA-biotin	S-67	Galβ1-3(GlcNAcβ1-6)GalNAcα-PAA-biotin
S-23	Galβ1-3GalNAcβ-PAA-biotin	S-68	Blood type A (tri)-PAA-biotin, sp=$(CH_2)_3NHCO(CH_2)_5NH$-
S-24	Galα1-3GalNAcβ-PAA-biotin	S-69	Blood type B (tri)-PAA-biotin, sp=$(CH_2)_3NHCO(CH_2)_5NH$-
S-25	Galβ1-3Galβ-PAA-biotin	S-70	GlcNAcβ1-3Galβ1-4GlcNAc-PAA-biotin
S-26	GlcNAcβ1-3Galβ-PAA-biotin	S-71	Neu5Acα2-3Galβ1-3GalNAcα-PAA-Biotin
S-27	αLacNAc-PAA-biotin	S-72	GlcNAcβ1-3(GlcNAcβ1-6)GalNAcα-PAA-biotin
S-28	Glcα1-4Glcβ-PAA-biotin	S-73	Galα1-4Galβ1-4GlcNAcβ-PAA-biotin
S-29	Galβ1-3GalNAcα-PAA-biotin, sp=-p-OC_6H_4-	S-74	GlcNAcβ1-4GlcNAcβ1-4GlcNAc-PAA-biotin
S-30	Galα1-2Galβ-PAA-biotin	S-75	Galβ1-3(Neu5Acα2-6)GalNAcα-PAA-biotin
S-31	GlcNAcβ1-4GlcNAc-PAA-biotin	S-76	Neu5Acα2-3(Neu5Acα2-6)GalNAc-PAA-biotin
S-32	GlcNAcβ1-4GlcNAcβ-PAA-biotin, sp=-$NHCOCH_2NH$-	S-77	Galβ1-4GlcNAcβ1-3GalNAcα-PAA-biotin
S-33	Neu5Acα2-6GalNAc-PAA-biotin	S-78	Le^b-PAA-biotin
S-34	H(type 3)-PAA-biotin	S-79	Le^y-PAA-biotin
S-35	3-HSO_3-Galβ1-4GlcNAc-PAA-biotin	S-80	Sialyl Le^a-PAA-biotin
S-36	3-HSO_3-Galβ1-3GlcNAcβ-PAA-biotin	S-81	Sialyl Le^x-PAA-biotin
S-37	Galα1-6Glcβ-PAA-biotin (melibiose)	S-82	GlcNAcβ1-3(GlcNAcβ1-6)Galβ1-4Glcβ-PAA-biotin
S-38	Neu5Acα2-8Neu5Acα-sp**-PAA-biotin, $(Neu5Ac)_2$	S-83	Galα1-3(Fucα1-2)Galβ1-4GlcNAc-PAA-biotin
S-39	Galβ1-2Galβ-PAA-biotin	S-84	Galβ1-3GlcNAcβ1-3Galβ1-4Glcβ-PAA-biotin
S-40	6-HSO_3-Galβ1-4GlcNAc-PAA-biotin	S-85	Galβ1-4GlcNAcβ1-3Galβ1-4Glcβ-PAA-biotin
S-41	Neu5Acα2-3Gal-PAA-biotin	S-86	(NeuAcα2-8)5-6-PAA-biotin
S-42	Galβ1-4(6-HSO_3)GlcNAcb-PAA-biotin	S-87	Galβ1-4GlcNAcβ1-3(Galβ1-4GlcNAcβ1-6)GalNAcα-PAA-biotin
S-43	3-HSO_3-Galβ1-3GalNAcβ-PAA-biotin (sulfate-TF)	S-88	α2-6 sialylated diantennary N-glycans-PAA-biotin
S-44	GlcNAcβ1-3GalNAcα-PAA-biotin	S-89	GalNAc-α-Ser-PAA-biotin
S-45	GlcNAcβ1-6GalNAcα-PAA-biotin	S-90	H_2O

α2-6 sialylated diantennary N-glycans :$(NeuAcα2\text{-}6Galβ1\text{-}4GlcNAcβ1\text{-}2Man)_2$α1-3,6Manβ1-4GlcNAcβ1-4GlcNAc

1

Supplementary information Figure 1

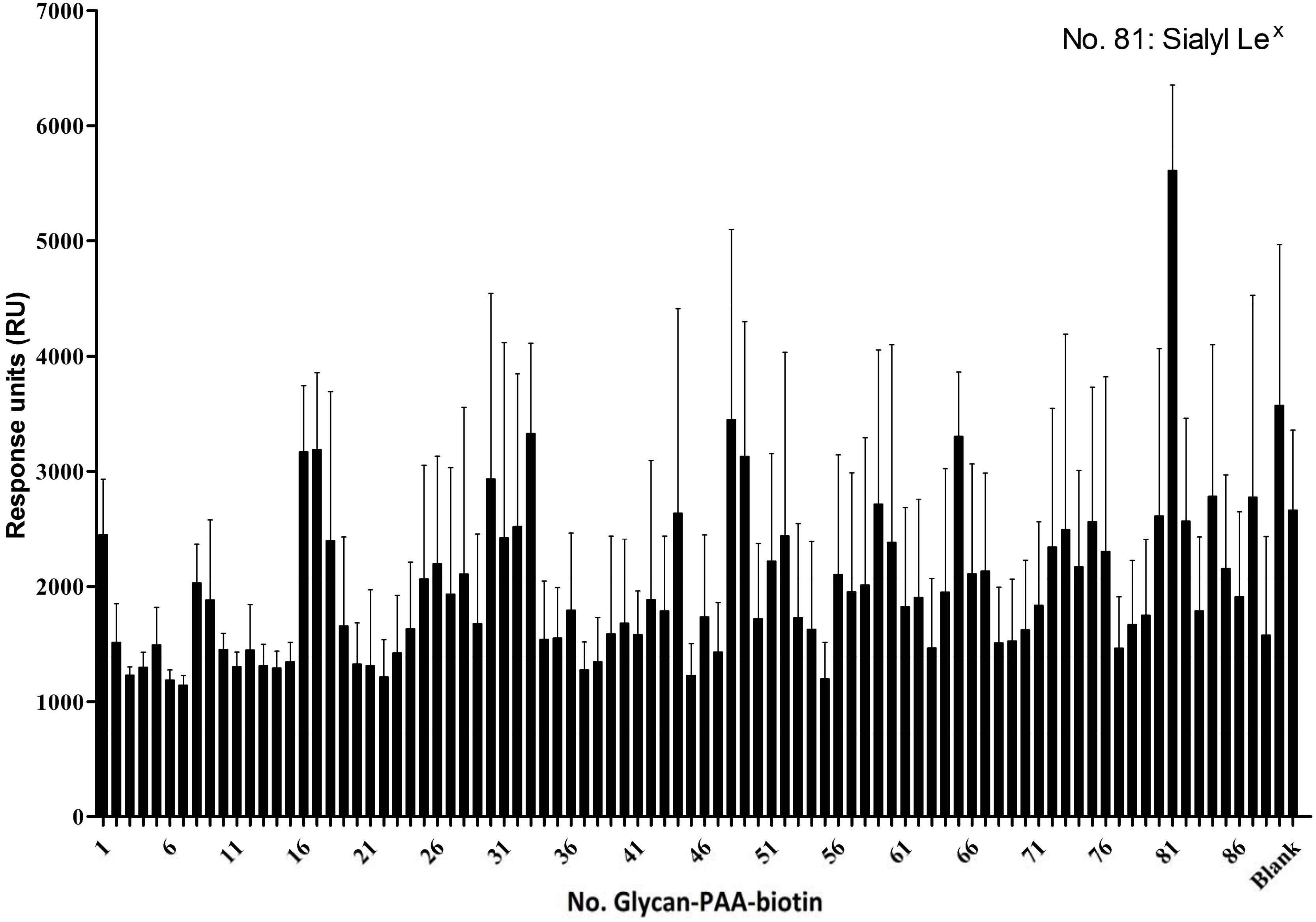

Supplementary information Figure 2

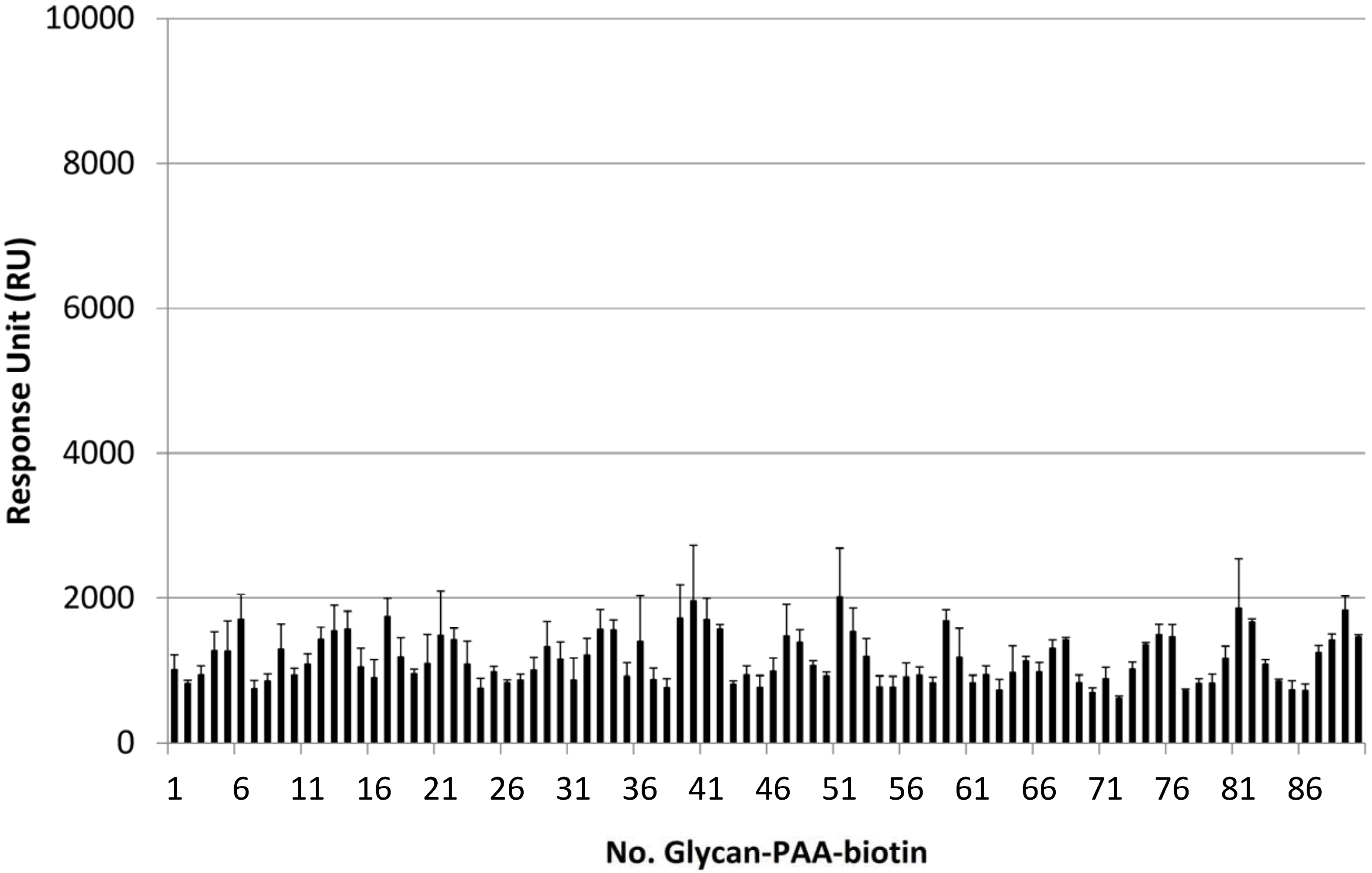

Supplement: Additional file 1 — Supplementary information. [file 1471-2180-12-162-S1.pdf]
